# Beware of Bear? Long-Term Spatio-Temporal Patterns of Human–Bear Conflict in Connecticut

**DOI:** 10.1007/s00267-024-02094-x

**Published:** 2024-11-30

**Authors:** Zachary Berkowitz, Larissa Montas Bravo, Shouraseni Sen Roy

**Affiliations:** 1 Abess Center for Ecosystem Science and Policy, University of Miami, Coral Gables, FL USA; 2 School of Geography and the Environment, University of Oxford, Oxford, UK; 3 Department of Research Data & Open Scholarship, University of Miami Libraries, University of Miami, Coral Gables, FL USA; 4 Department of Geography & Sustainable Development, University of Miami, Coral Gables, FL USA

**Keywords:** Bears, Emerging hot spots analysis, Human–bear conflict, Forest-based and boosted classification regression, Connecticut

## Abstract

In this study, we examine the spatio-temporal patterns of citizen-reported human–bear conflict (HBC) from 2002 to 2022 and use the Forest-Based and Boosted Classification (FBBC) technique to assess the significance of several factors in the occurrence of HBC. Our analysis reveals a significant increase in HBC incidents over the study period, with the fewest conflicts in 2002 (217) and the most in 2022 (4455). These were concentrated in northwestern Connecticut, particularly eastern Litchfield County and western Hartford County. The results of geostatistical analysis, including measures of dispersion and emerging hot spot analysis indicated a southward trend in HBC on both annual and monthly scales. The validation results of the FBBC highlighted the relevance of forest fragmentation, intermediate housing density, proximity to water bodies, and snowfall in predicting HBC. Each variable demonstrated nearly equal importance (20%) in predicting HBC occurrences from 2010 to 2022, though land cover showed no significant predictive power. These findings elucidate the spatio-temporal dynamics of HBC and offer valuable insights for wildlife managers to prioritize conflict mitigation strategies effectively. The results of this study identify locations prone to HBC. Moreover, FBBC results show that this technique can be used to predict future HBC based on projected changes in these variables due to climate change and expansion of the human–wildlife interface. Our analysis can aid in the development of targeted, evidence-driven, and ethical management interventions in Connecticut.

## Introduction

Scientific literature and reports indicate human–wildlife conflicts (HWC) are increasing around the world, often due to population growth and the expanding human–wildland interface (Messmer 2000, 2009; Henle et al. 2008; White and Ward 2010). The term HWC describes instances where wildlife encroaches on human settlements, posing threats to human recreational activities, life, and economic well-being (Dickman 2010; Soulsbury and White 2015; Nyhus 2016). It can also extend to indirect impacts, such as affecting psychosocial well-being (Barua et al. 2013; Nyhus 2016; Blackie 2023). Historically, human responses to HWC have involved the lethal removal of “problem” or “nuisance” wildlife, sometimes leading to species extinctions or significant population declines (Woodroffe et al. 2005; Treves et al. 2006; Dalerum et al. 2009; Conover and Conover 2022). In much of the eastern United States, black bears (*Ursus americanus*) are implicated in HWC. Although black bears seldom attack humans, they are known to prey on livestock, scavenge for garbage, and even enter homes in search of food (Spencer et al. 2007; Herrero 2018; Smith and Herrero 2018). With their imposing stature and powerful bite, the mere presence of black bears is often perceived as a threat to public safety.

During the 1800s, black bears experienced severe population and range loss across North America due to overhunting and habitat loss (Hellgren and Maehr 1992; Scheick and McCown 2014; Clark et al. 2021). Nonetheless, in some locations, such as Connecticut, black bears have made a comeback (Garshelis and Hristienko 2006; Scheick and McCown 2014; Evans et al. 2017). Anthropogenic food sources, such as garbage and bird seed, have facilitated the recolonization of black bears (Merkle et al. 2013; DeStefano and DeGraaf 2003; Evans et al. 2017; Johnson et al. 2015), thereby increasing human–bear conflict (HBC) (Baruch-Mordo et al. 2008; Beckmann and Lackey 2008; Greenleaf et al. 2009; Evans et al. 2014; Obbard et al. 2014; Wilton et al. 2014; Lewis et al. 2015). A 2007 survey of North American wildlife agencies found that garbage and other food attractants were the most common form of HBC (Spencer et al. 2007). As opportunistic omnivores with significant behavioral adaptability and social learning capacity, black bears can quickly adapt to varying conditions (Beckmann and Berger 2003) and may be regarded as synanthropes (Gilbert 1989; Mazur and Seher 2008). Responses to the urban–wildland interface and HBC have shown to vary seasonally and temporally, with bears in some areas becoming more active at night to avoid detection (Ranglack et al. 2009; Baruch-Mordo et al. 2014; van Bommel et al. 2022).

Furthermore, climate change can initiate or accelerate such changes in black bear ecology that result in conflict. For instance, when masting plant species, such as those that create berries fail, black bears often turn to anthropogenic food sources (Baruch-Mordo et al. 2008; Baruch-Mordo et al. 2014; Lewis et al. 2015). Ahead of winter, hyperphagic black bears demand a substantial food intake. Recognizing the reliability of anthropogenic food sources compared to unpredictable wild crops, these bears may increasingly engage in HBC (Lewis et al. 2015; van Bommel et al. 2022). Despite not being true hibernators, black bears undergo physiological changes during the winter to cope with reduced food availability and cold temperatures, such as a slower metabolism, decreased heart rate, and slightly lowered body temperature (Hellgren 1998; Tøien et al. 2011; Kirby et al. 2019; DEEP 2024a). Some bears will remain active during periods of the winter if there is little snow cover and higher-than-usual temperatures, indicating that climate change could exacerbate HBC (Baruch-Mordo et al. 2014; Johnson et al. 2018). Bears who forage more on anthropogenic food sources have also exhibited shorter hibernation periods (Johnson et al. 2018; Kirby et al. 2019).

Landscapes characterized by intermediate housing density provide the optimal habitat for bears seeking convenient access to food (Kretser et al. 2008; Evans et al. 2014; van Bommel et al. 2020). These landscapes offer abundant edge habitats where bears can seek shelter when not foraging on anthropogenic food sources (Kindal and van Manen 2007; Baurch-Mordo et al. 2008; van Bommel et al. 2020; Boudreau et al. 2021). Other natural habitat variables also correlate with HBC. Numerous studies focusing on the landscape-scale habitat selection of black bears have confirmed their preference for riparian habitat, increasing HBC in such areas (Clark et al. 1993; Merkle et al. 2011). Nevertheless, black bears exhibit a reluctance to inhabit urban areas (Baruch-Mordo et al. 2014; Johnson et al. 2015; Evans et al. 2017). Although black bears exhibit an acute awareness of immediate anthropogenic threats, mortality rates are higher among bears exploiting human settlements, often because of roadkill incidents (Beckmann and Lackey 2008; Lamb et al. 2016; van Bommel et al. 2022). A host of studies have demonstrated that roads elicit behavioral responses in black bears (Ditmer et al. 2018), ranging from attraction to high-quality forage sites along roads (Lewis et al. 2011) to avoidance of highly trafficked roads (Beringer et al. 1990; McCown et al. 2009).

Connecticut is one such state facing high rates of HBC, as the growing black bear population recolonizes the state. About five studies have examined Connecticut’s bear population, however, none of the studies incorporated data post-2019 (Evans et al. 2014, 2017, 2018a, b, 2019), and only one focused on HBC (Evans et al. 2014). A study by Evans et al. (2014), which examined HBC from 2008 to 2012, found that certain factors help predict HBC: increased forest edge density, intermediate percent forest cover, intermediate distance to wetlands, and proximity to streams. Evans et al. (2017) found that in intermixed landscapes bear densities correlate more strongly with housing density than forest cover. These studies emphasize that exurban landscapes (6–49 homes/km^2^) with homes interspersed within forest are associated with the highest rates of HBC, particularly in the northwestern region of Connecticut (Evans et al. 2014, 2017). In fact, peak bear density was observed where housing density was 7.5–18.2 homes/km^2^. Urban areas were associated with the lowest rates of black bears (~0 when housing density was 35–50 homes/km^2^) (Evans et al. 2017). Exurban landscapes also promote the recolonization of black bears, once extirpated from the state (Evans et al. 2017). Connecticut’s largely exurban landscape is considered 72% intermixed, seemingly offering the perfect habitat for recolonization, and unfortunately HBC. The Connecticut Department of Energy and Environmental Protection (DEEP) and other published studies emphasize the bear population has primarily expanded west of the Connecticut River, which runs through the center of the state and the area of highest development. Evans et al. (2017) asserted that the bear population is “effectively bounded by urbanization along the Connecticut River,” but may recolonize southeastern Connecticut in the future (Evans, p. 18).

The DEEP asserts that rising rates of HBC in Connecticut are not due to habitat loss, but rather attraction to anthropogenic food sources (DEEP 2024c). An estimated 1000–1200 bears live in the state, with males growing up to 550 lbs and females reaching 300 lbs (DEEP 2024a). The growth and range expansion of the bear population has even garnered significant attention from lawmakers. During the 2023 legislative session, a bill proposing 50 bear hunting permits was introduced. The bill failed within the environmental committee due to strong opposition from a coalition of environmental and animal advocacy organizations (Moritz 2023; Raff and Sobol 2023). In June 2023, Governor Ned Lamont signed a less comprehensive bill resembling a “stand-your-ground” law for bears. Under this law, Public Act 23-77, Connecticut residents can shoot and kill bears that pose an imminent threat to them or their pets. The law also authorizes the DEEP commissioner to grant kill permits to landowners whose crops, livestock, or apiaries have been damaged. The law does not prohibit unintentional feeding of bears, one of the major provisions in the original bill (Moritz 2023; Spiegel 2023). However, it does prohibit intentional feeding of bears (DEEP 2024b).

Despite a growing body of literature on black bears, little is known about how HBC varies spatially or temporally in areas where they are recolonizing. Likewise, while predictive models of HBC have been developed, few give a holistic illustration of HBC on a statewide scale in an area of bear recolonization. Most of these studies focus on black bears in rural areas, where livestock predation is the most common form of HBC (Wilson et al. 2006; Baruch-Mordo et al. 2008). Therefore, we analyzed the spatio-temporal patterns of HBC using long-term citizen-reported data from 2002 to 2022. In addition, by leveraging ancillary datasets, such as forest fragmentation data, we elucidate how anthropogenic development and land use change have affected these patterns. To reduce HBC, wildlife managers must have sufficient knowledge not only of HBC hot spots, but areas with a high probability of HBC in the future (Sitati et al. 2003; Wilson et al. 2006; Kretser et al. 2008). The results of our research revealed the extent of the bear population’s growth and expansion in Connecticut, thus informing wildlife managers of the most important areas to target for conflict mitigation strategies. Provided there is sufficient public awareness about an accessible reporting mechanism like Connecticut’s, our methods can be replicated in other regions to develop informed, targeted conflict mitigation programs.

## Data

### HBC Data

**Fig. 1:**
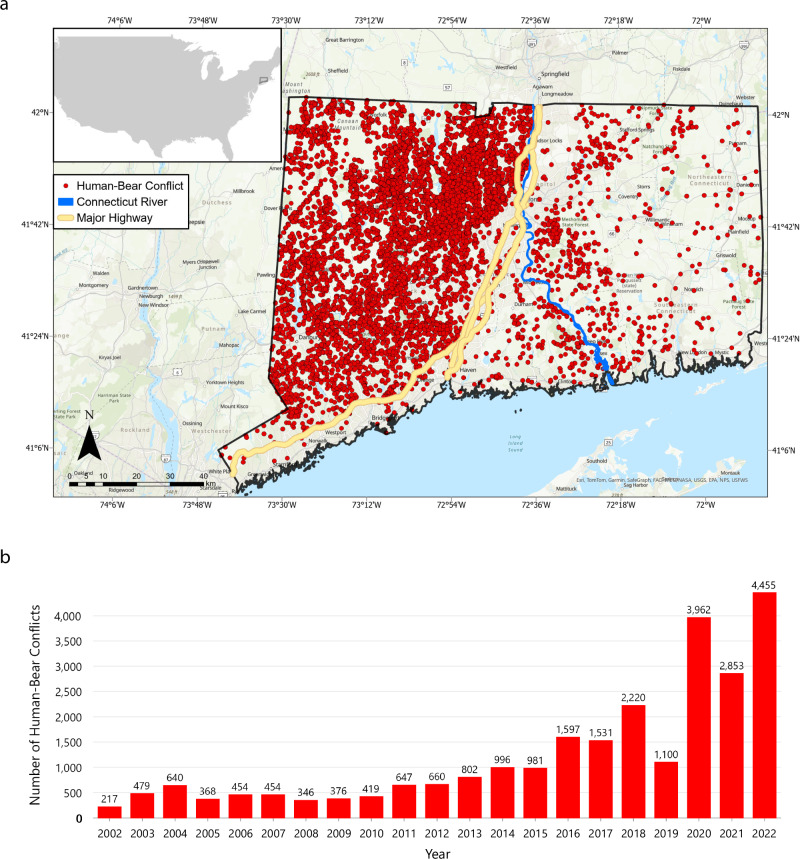
**a** Citizen-reported human–bear conflict (HBC) in Connecticut from 2002 to 2022. **b** Annual citizen-reported HBC in Connecticut from 2002 to 2022

The DEEP provided citizen-reported data on bear and bobcat sightings spanning from 2001 to 2022. The DEEP has extensive, easily accessible informational resources on their website for residents to report bear sightings/HBC and understand the dynamics of living with black bears (Connecticut Department of Energy and Environmental Protection (DEEP) 2024a, b, c). Some records in the dataset are from the DEEP itself, such as in cases where environmental conservation police officers responded to HBC emergencies. Our analysis focused specifically on the bear data, excluding the year 2001. This exclusion is to accommodate for a potential delay in public awareness regarding the reporting of sightings. The total number of records for bear sightings was 93,976, of which 25,557 records were HBC (Fig. 1). This dataset has been widely used to examine bear sightings and HBC in Connecticut.

### Ancillary Data

**Fig. 2:**
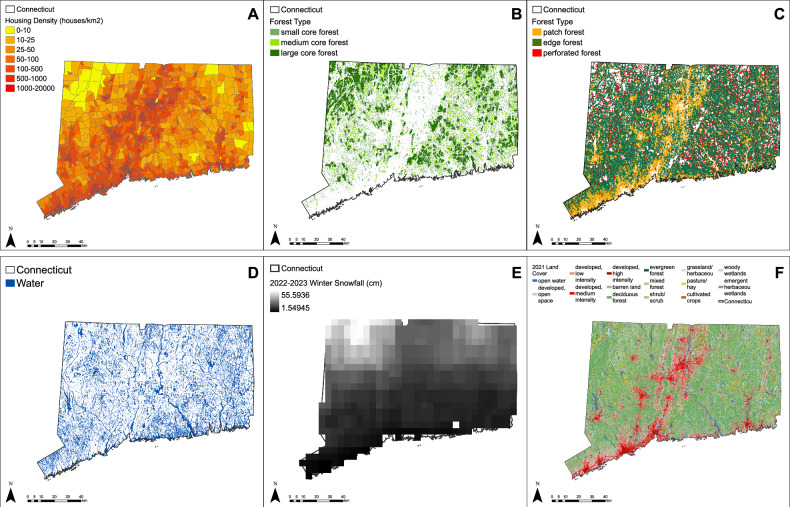
Spatial distribution of ancillary data **A** 2020 Housing density (houses/km^2^), **B** 2015 core forest, **C** 2015 fragmented forest, **D** hydrography, **E** 2022–2023 snowfall (cm), and **F** 2021 land cover in Connecticut

To create a predictive model of the spatial spread of HBC in Connecticut, we utilized six causal variables: housing density (houses/km^2^), distance to fragmented forest (m), distance to core forest (m), distance to water (m), land cover, and seasonal snowfall (cm) (Fig. 2). Housing density data for 2010 and 2020 were obtained from the U.S. Census Bureau (U.S. Census Bureau, 2010a, b, 2020a, b). The data were provided as the number of housing units per block group, from which we calculated density/km^2^. Housing density ranged from 0 houses/km^2^ to 16,203 houses/km^2^, with the latter found in a Stamford block group (Fig. 2A).

Forest fragmentation data for 2010 and 2015 were downloaded from the University of Connecticut’s Center for Land Use Education and Research (UConn CLEAR 2015). This dataset delineates seven classes: non-forested, patch forest, edge forest, perforated forest, small core forest, medium core forest, and large core forest. Derived from National Aeronautics and Space Administration Landsat imagery with a spatial resolution of 30 m, the accuracy assessment conducted by UConn CLEAR yielded 82.18% accuracy for the 2010 data and 81.82% accuracy for the 2015 data, based on the primary reference point. We classified the forest data into “fragmented forest,” encompassing patch, edge, and perforated forest, and “core forest,” comprising small core, medium core, and large core forest. Edge effects, defined as “abrupt changes in vegetative populations or community structures found at the boundary of two or more different habitats,” influence the degradation of patch, edge, and perforated forest. The area of core forests almost remained the same at 3839.76 km^2^ in 2010 to 3846.65 km^2^ in 2015 (Fig. 2B). Fragmented forest area slightly decreased from 3696.19 km^2^ in 2010 to 3689.07 km^2^ in 2015 (Fig. 2C). The distance of HBC to fragmented forest peaked at 866.22 m, while distance to core forest reached a maximum of 4796.31 m in 2015.

To determine distances to water, we used a hydrography polygon shapefile from the DEEP, derived from the U.S. Geological Survey data (Connecticut Department of Energy and Environmental Protection (DEEP) 2019c). The hydrography data, initially compiled in 1994 by the USGS and subsequently updated in 2005 for enhanced usability, encompass various water-related features, including marshes (Fig. 2D). Distance to water peaked at 1630.08 m and we used a minimum distance of 0.30 m for HBC records overlapping with water, likely due to the use of inaccurate GPS reporting systems.

Seasonal snowfall data for each year of the study (2010–2022) was acquired from the National Operational Hydrologic Remote Sensing Center (NOHRSC) (NOHRSC 2023). The NOHRSC data, featuring a spatial resolution of 1 km and a temporal resolution of 24 h, amalgamates various observations, including those from National Weather Service spotter observers. This data serve as a proxy for hibernation length and bear activity levels during winter. Further information about the data can be found at NOAA (2024). Snowfall peaked at 214.73 cm during the 2010–2011 winter season in the northwestern corner of the state (Fig. 2E).

Land cover data with a 30-m spatial resolution was extracted from the National Land Cover Database, covering years 2011, 2013, 2016, 2019, and 2021 (MRLC 2021). Connecticut exhibits 15 land cover classes, with deciduous forest predominating at 51.33% coverage in 2021 (Fig. 2F). Mixed forest and developed open space follow as the second and third most predominant classes, at 13.39% and 9.92%, respectively.

Finally, administrative boundaries data at the county and state level were obtained from ArcGIS Online while data on town boundaries and major transportation roadways were acquired from the DEEP website (Connecticut Department of Energy and Environmental Protection (DEEP) 2019a, b).

## Methods

**Fig. 3:**
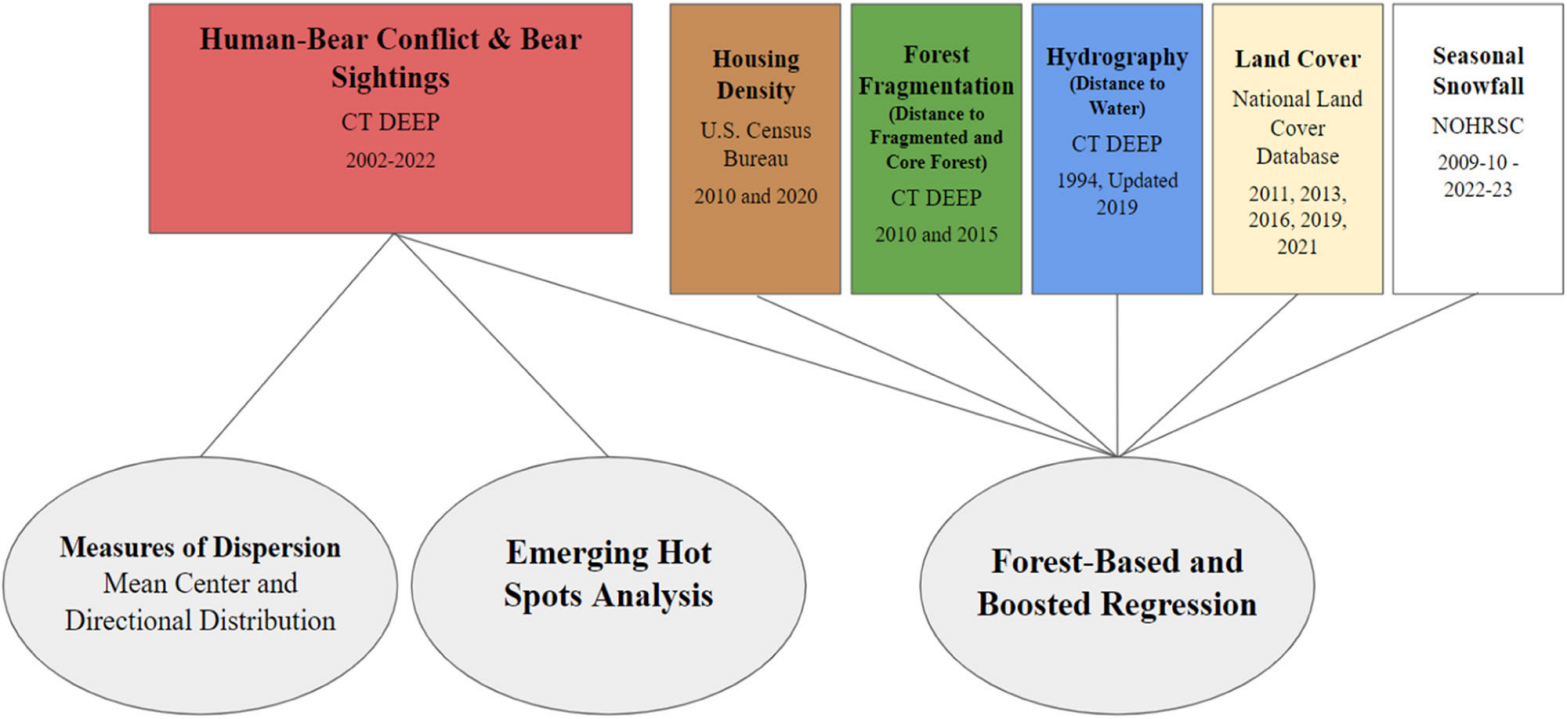
Outline of research methods

The outline of research methods and the various datasets used in this study are shown in Fig. 3. All spatial data were projected onto the same coordinate system as the HBC data: NAD 1983 State Plane CT FIPS 0600 Feet using ArcGIS Pro. We first refined the bear sightings dataset, deleting a small number of records found from 1986 to 2001, notwithstanding the DEEP’s claim that the database contained records from 2001 to 2022. Records with “<Null>” in the “date and time of sighting” field were also deleted as well as records found outside the state border. According to the Connecticut Department of Energy and Environmental Protection (DEEP) (2024a), black bear litter sizes range from one to five in the state of Connecticut, therefore records with more than six bears reported were deleted. One record with “−1” bears was deleted, although the comments indicated that there was one bear being reported. To account for the potential delay in the public’s awareness regarding their ability to report bear sightings, we excluded data from 2001. This left us with 93,976 bear sightings.

**Fig. 4:**
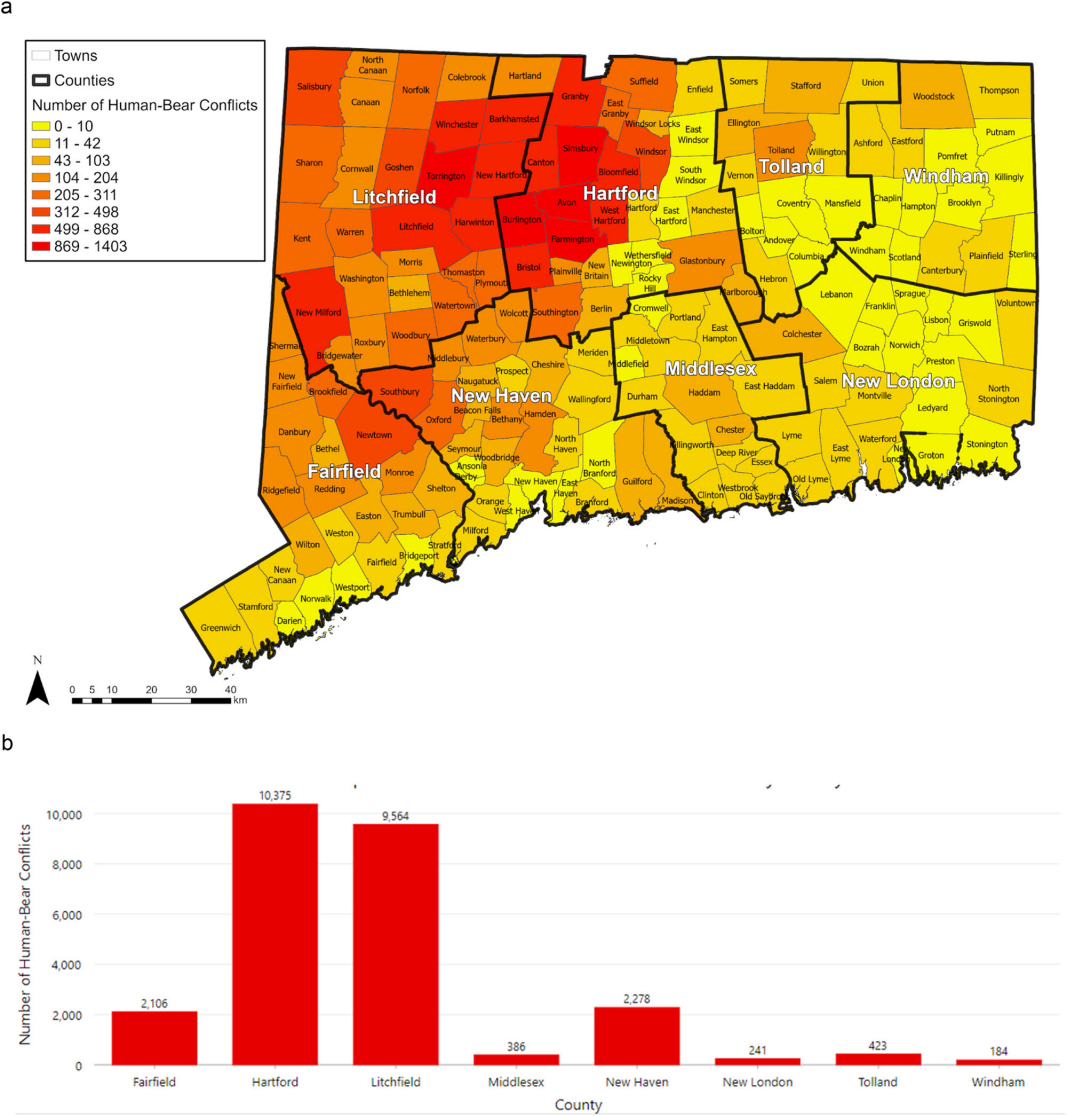
**a** Citizen-reported HBC from 2002 to 2022 by town. **b** Citizen-reported HBC from 2002 to 2022 by county

We then identified records of HBC among the bear sightings, defined as any human–bear interaction that leads to tangible harm to either bears, humans, or their belongings, including but not limited to pets, livestock, and waste materials. This definition is similar to one of the most extensive definitions of “bear incident” developed by Hopkins et al. (2010). Specifically, all records where the type of sighting was equal to roadkill were identified as HBCs. In addition, after detailed examination of the information recorded under “Additional comments” field, a list of 34 search terms, including nuisance, raid, and birdfeeder, were identified as evidence of HBC. We also checked in detail the “Further comments about damage or incident” field to make sure that non-HBC bear sightings were not included in the final analysis. Based on the criteria for identifying HBC, the final number analyzed included 25,557 incidents, including 22,223 incidents from 2010 to 2022 (Fig. 4).

### Measures of Dispersion

**Fig. 5:**
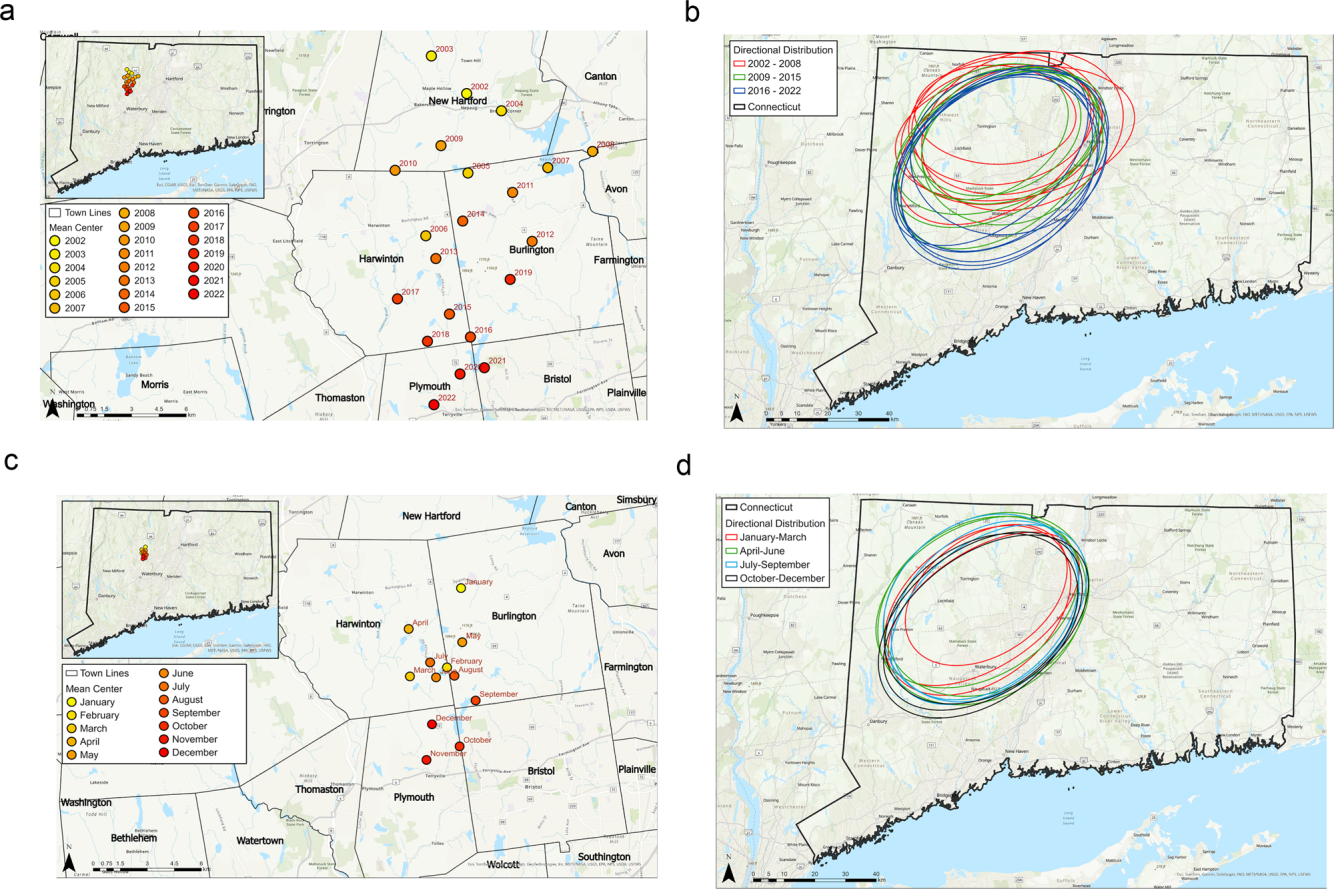
Measures of dispersion of citizen-reported HBC in Connecticut from 2002 to 2022. **a** Annual mean centers. **b** Annual directional distribution standard deviation ellipses. **c** Monthly mean centers. **d** Monthly directional distribution standard deviation ellipses

We analyzed the spatial distribution of the HBC using several geostatistical measures of dispersion, including mean centers and standard deviational ellipses (SDE). The mean centers were calculated using the average of the *x* and *y* coordinates for all the locations of HBC at the annual and monthly scale (Wright and Sen Roy 2022). The change in location of the mean centers is useful for identifying changes in spatial patterns of HBC from 2002 to 2022 (Fig. 5a, c). Given the year-to-year variations in HBC, we also calculated the SDE to determine the directional trend and variation at the annual and monthly scale by calculating the standard deviation of the *x* and *y* coordinates from the mean centers to define the axis of the ellipse (ESRI 2024a). The specific calculations for the SDE are given below:

$$C=\left(\begin{array}{cc}{var}\left(x\right) & {cov}\left(x,y\right)\\ {cov}\left(y,x\right) & {var}\left(y\right)\end{array}\right)=\frac{1}{n}\left(\begin{array}{cc}\mathop{\sum }\limits_{i=1}^{n}{\widetilde{x}}_{i}^{2} & \mathop{\sum }\limits_{i=1}^{n}{\widetilde{x}}_{i}{\widetilde{y}}_{i}\\ \mathop{\sum }\limits_{i=1}^{n}{\widetilde{x}}_{i}{\widetilde{y}}_{i} & \mathop{\sum }\limits_{i=1}^{n}{\widetilde{y}}_{i}^{2}\end{array}\right){\rm{where}}$$

$$\begin{array}{l}{\mathrm{var}}\left(x\right)=\frac{1}{n}\mathop{\sum }\limits_{i=1}^{n}{\left({x}_{i}-\widetilde{x}\right)}^{2}=\frac{1}{n}\mathop{\sum }\limits_{i=1}^{n}{\widetilde{x}}_{i}^{2}\\{\mathrm{cov}}\left(x,\,y\right)=\frac{1}{n}\mathop{\sum }\limits_{i=1}^{n}\left({x}_{i}-\widetilde{x}\right)\left({y}_{i}-\widetilde{y}\right)=\frac{1}{n}\mathop{\sum }\limits_{i=1}^{n}{\widetilde{x}}_{i}{\widetilde{y}}_{i}\\\quad\,\,{\mathrm{var}}\left(y\right)=\frac{1}{n}\mathop{\sum }\limits_{i=1}^{n}{\left({y}_{i}-\widetilde{y}\right)}^{2}=\frac{1}{n}\mathop{\sum }\limits_{i=1}^{n}{\widetilde{y}}_{i}^{2}\end{array}$$

where *x* and *y* are coordinates for each HBC location, *{*${\bar{x}},\, {\bar{y}}$*}* represents the annual mean centers of HBC, and n is equal to the total number of features (ESRI 2024a). The spatial patterns of the annual SDE are shown in Fig. 5b, d.

### Emerging Hot Spots Analysis (EHSA)

**Fig. 6:**
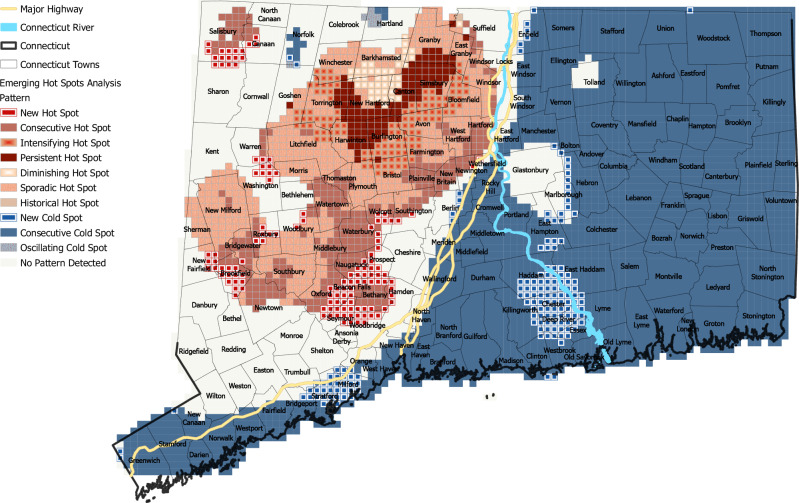
Results of emerging hot spots analysis of HBC in Connecticut from 2002 to 2022

In view of the substantial variations in the total number and spatial variation in the distribution of HBC over time, we utilized Emerging Hot Spots Analysis (EHSA). This process consists of two steps: (a) a space-time cube, and (b) the actual analysis. The space-time cube is a netCDF file containing the location *x, y* coordinates, with a *z* coordinate for time. The time step interval for the space-time cube was set to 1 year. The space-time bins are next used to calculate hot spot trends among the cubes of data within the study area using the Getis-Ord Gi* statistic, thus categorizing them into hot or cold spot variations (Tullis-Joyce and Sen Roy 2021). Once the space-time hot spot analysis is completed, then each bin in the input netCDF cube gets assigned a z-score, *p* value, and hot or cold spot bin classification. The conceptualization of the relationship for the analysis was set to fixed distance. Next, these hot and cold spot trends are evaluated using the Mann–Kendall trend test (ESRI 2024b). The EHSA tool categorizes the data cubes into eight hot or cold spot pattern types, using the calculated trend z-score and *p* value for each location in the data, along with the hot spot z-score and *p* value for each bin (Fig. 6). The pattern types (excluding “no pattern detected”) are as follows: new, consecutive, intensifying, persistent, diminishing, sporadic, oscillating, and historical. New hot and cold spots are the locations that are statistically significant hot or cold spots only during the final time-step interval, in our case the final year of analysis 2022, and none before. Consecutive hot or cold spots are locations that experienced a run of at least two statistically significant hot or cold spot bins in the final time-step interval but were never statistically significant hot or cold spots before the last time step (Gale and Sen Roy 2023). Intensifying, persistent, and diminishing hot or cold spots were all statistically significant hot or cold spots for at least 90% of the time steps, including the final time step. However, intensifying refers to a statistically significant increase in clustering over time, contrasted with a decrease in clustering in diminishing hot/cold spots, and no discernible trend with persistent hot/cold spots. The sporadic hot and cold spots include locations that are statistically significant hot or cold spots for the final time-step interval, with less than 90% of the time step being statistically significant hot or cold spots. Also, the opposite pattern was not exhibited in any of the prior time steps. Therefore, none of the time steps in a sporadic hot spot would have been statistically significant cold spots. Oscillating hot/cold spots are the same, but the opposite pattern was detected during a previous time step. Accordingly, some of the time steps prior to the last one would have been statistically significant cold spots. The historical hot/cold spot pattern type indicates at least 90% of the time-step intervals were statistically significant hot/cold spots, but not the last one.

### Forest-Based and Boosted Classification (FBBC)

The random forest (RF) algorithm is a machine learning ensemble method that can be applied on spatial data to solve problems with data classification, and prediction requirements. Breiman (2001) first developed the RF algorithm by integrating a bagging sampling approach (Breiman 1996), random split selection (Dietterich 1998), and various processes for random feature selection (Ho 1995, 1998; Amit and Geman 1997; Fawagreh et al. 2014). Both dependent and independent variables can be categorical or numerical. While the RF algorithm can be used to model regression of continuous numerical variables, it mainly performs well as a classification model for categorical dependent variables, for example, whether human–bear conflict occurred or not during a bear sighting. When the dependent variable is categorical, the algorithm assesses the output of multiple decision trees, trained on subsets of randomly selected independent variables and randomly selected parts of the same training dataset, with the goal of overcoming the overfitting problem of individual decision trees. This method constitutes an ensemble learning method for classification, which constructs a large number of decision trees in training mode and outputs the class that is the mode of all the classes output by individual trees. This approach is commonly known as a “majority voting” given the output that receives the largest number of “votes” is selected as the final classification decision. The majority wins methods have lower bias and prediction variance compared to predictions from individual decision trees. Thus, the RF classifier relies on an ensemble of single classification trees and is well suited for high-dimensional data and multiclass problems.

We used the FBBC method to classify the bear sightings records into sightings where HBC occurred and sightings that were not associated with HBC. We used data from 2010 to 2022 for this part of the analysis due to the availability of complete data for all the independent variables to do a robust analysis. The model relied on six independent variables: distance to fragmented forest, distance to core forest, distance to water, snowfall, housing density, and land cover. All these variables were marked as continuous, except for land cover that was designated as a categorical variable. The dependent variable was the classification of HBC or no HBC at locations of citizen-reported bear sightings.

The first step involved using the FBBC algorithm to train a model by building a sequence of trees and establishing a relationship between the independent variables and the categorical dependent variables. All the 2010–2022 data were marked as training data. However, a random subset corresponding to 10% of the training data was excluded and marked as validation data. Once the decision trees were finalized, a second step involved evaluating model performance by using the training data to predict the dependent variable, compare the predicted category to the observed category and calculate diagnostics. This was conducted for both the training data and the validation data.

Model characteristics, model out-of-bag (OOB) errors, variable importance diagnostics, and classification diagnostics for the training data and the validation data were recorded.

## Results

Spatially, the distribution of the HBC showed a greater concentration in the western part of the state divided by three major highways (State Route 15, US Route 5, and Interstate 91) and the Connecticut River. The number of HBCs exhibited a significant increase from 2002 to 2022. The lowest recorded instances of HBC (217) were documented in 2002, contrasting sharply with the highest tally (4455) reported in 2022 (Fig. 1b). Despite a clear increasing trend, the data also revealed fluctuations, notably with a decrease by at least 1000 incidents in 2019 and 2021. Over the span of 2002 to 2022, a total of 93,976 bear sightings were reported to the DEEP, out of which 25,557 incidents were HBC.

We observed a distinct concentration of HBC in eastern Litchfield County (9564 cases) and western Hartford County (10,375 cases) (Fig. 4a). None of the other counties reported more than 2500 HBC incidents. The town of Avon alone accounted for 5.49% (1403) of the total HBC incidents in Connecticut. It was followed by Simsbury (1383), Torrington (1306), Farmington (1124), and Burlington (983). Conversely, Windham County experienced the fewest HBC incidents (184). While all the towns reported bear sightings, only three out of 169 towns did not experience any HBC incidents during the study period: West Haven, New London, and Franklin. Additionally, the towns of Scotland, Preston, Bridgeport, and Darien recorded only one incident each.

The analysis of HBC-related causes indicated about 37.74% (9646 out of a total 25,557) of the total incidences involved garbage. Torrington exhibited the highest number of garbage-related HBC (722), ahead of Simsbury (610), and Avon (540). Most of the towns (136 of 169 towns) reported a garbage-related HBC, and 2.29% (586) of the HBCs consisted of roadkill incidents that occurred in 82 towns. Winchester reported the most roadkill incidents (37), followed by Litchfield (34) and Granby (32). In addition, there were 248 reports of bear home entries during the entire study period, of which 144 occurred in the last 3 years of the study period (2020–2022). From 2002 to 2009, only five bear home entries were reported. Salisbury recorded the highest number of bear home entries (30), followed by Canton (28), and Norfolk (17). At the county level, most of the home entries were concentrated in Litchfield and Hartford County, with seven incidents reported in Fairfield County and one in New Haven County. The rest of the counties did not report any bear home entries.

Given the substantial variations in the spatio-temporal patterns of HBC, we next analyzed the measures of dispersion at the annual and monthly scale. The annual mean centers showed a southward trend in HBC. The mean centers for 2002–2004 were located in New Hartford, which moved just south of New Hartford in 2005. The southward trend of mean centers continued through Burlington (2005–2007, 2009, 2011–2016, 2019) into southeastern Harwinton (2017), northwestern Bristol (2021), and northeastern Plymouth (2018, 2020, and 2022). The annual SDE, indicative of directional trends, reflected a similar pattern, trending in a northeast-to-southwest direction over time. The SDE for the earlier years (2002–2008) also showed a stronger east-west orientation, while it changed to more north-south direction in later years (2016–2022). Furthermore, the overall size of the SDE increased with time, indicating wider spatial spread over time.

At the monthly scale, the mean centers for the warmer months, April through August, were distributed in a more random pattern in Harwinton and Burlington. Conversely, the northernmost location for the mean centers in Burlington was noted in January. Nevertheless, the mean centers for the colder months, September through December, were concentrated in southern Burlington and northern Plymouth. This can be attributed to the seasonality and less activity of bears in the north where it is cooler. The monthly SDE exhibited a southwest trend in HBC, being more compact in the earlier months as well. We observed little difference in the SDE between April and September.

Due to the substantial spatial variations in the measures of dispersion and a southward trend over time, we utilized EHSA to reveal the trends in spatial patterns of HBC over the study period (Fig. 6). The results of the EHSA analysis revealed a distinct divide in the state of Connecticut along the three major highways (State Route 15, US Route 5, and Interstate 91) and the Connecticut River. Hot spots were observed throughout much of the state west of the highways, disregarding the shoreline. Cold spots covered most of the state east of these three major highways. The Connecticut River also appears to bound hot spots/cold spots north of Wethersfield, where it follows a similar path to the major highways. The entire coastline, stretching from Greenwich to Stonington, was classified as cold spots.

Sporadic hot spots were shown as the most prevalent form of hot spots, covering 1253.32 km^2^, extending from the Massachusetts–Connecticut border to as far south as Newtown. Most of Litchfield and New Milford, both located near the western border of Connecticut, were classified as sporadic hot spots. The second most prevalent form of hot spots was consecutive hot spots, covering 775.25 km^2^. These were concentrated just west of the aforementioned highways, in addition to patches in the northwestern corner of state and the Bridgewater, Brookfield, and Newtown areas. Intensifying hot spots extended across 333.36 km^2^, forming a contiguous concentration from western Bloomfield through significant parts of Simsbury, Avon, Burlington, Farmington, and Bristol. Citizen-reported HBC also intensified throughout much of Torrington and its neighboring towns. We observed 279.09 km^2^ of persistent hot spots in a nearly continuous concentration throughout southern Granby, Simsbury, Canton, southern New Hartford, northern Burlington, eastern Harwinton, and eastern Torrington. Diminishing hot spots were confined to one patch, primarily in Barkhamsted and New Hartford. New hot spots were identified in a disorganized distribution, with a significant number in central New Haven County and southern Litchfield County. We found one historical hot spot (2.58 km^2^) in Suffield along the border with Massachusetts (Fig. 6).

The various types of cold spots, indicative of declining trends over time, were most concentrated to the east of the three major highways and Connecticut River running through the center of the state. The most expansive pattern covering 6669.69 km^2^ was observed for consecutive cold spots, dominating the state east of I-91 Interstate and US Route 5. On the other hand, two notable patches devoid of discernible patterns were observed in the eastern half of the state: Glastonbury and Tolland. Two significant clusters of new cold spots emerged, one located in Stratford and Milford, and the other in Deep River, Chester, and Haddam. Only two localized cold spots, oscillating cold spots, were apparent in western Connecticut, specifically along the Norfolk–Goshen border and Hartland. No pattern was detected in 3201.75 km^2^ of the state, primarily surrounding the large clusters of hot spots in western Connecticut (Fig. 6).

In view of the significant clusters in hot and cold spots over time, we used FBBC algorithm to assess the relevance of identified potential causal factors and bear sightings resulting in HBC. This algorithm created 100 trees with a leaf size of one. The tree depth ranged from 5318 to 6814 with a mean tree depth of 5799. We excluded 10% of the training data for validation. Model OOB errors were quite similar when using 50 trees versus 100, but we focused on the model OOB calculated for 100 trees since it was a more accurate measure. These OOB errors were calculated using part of the training dataset that was not seen by a subset of the trees in the forest. There was an overall mean squared error of 32.21, 12.68 for non-HBC bear sightings (“No”), and 82.83 for HBC (“Yes”). Results for the Gini coefficient computations show that five of the six variables had a 20% importance rank. Distance to fragmented forest was the most important variable in determining HBC with an importance index of 1555.13. Distance to core forest was the second most important (1522.37), followed by distance to water (1513.98), housing density (1502.04), and snowfall (1500.81). Land cover had an importance rank of less than 0.01% and an importance index of 5.73. Therefore, the most parsimonious prediction run should exclude land cover. During the training run, the model sensitivity for the “no” HBC category was reported at 98% while sensitivity for the “yes” HBC category was reported at 26%. Thus, the model correctly predicted the “no” HBC category 98% of the time, and “yes” HBC category 26% of the time. Overall, the trained model was 70% accurate with a 63% F1-score.

After the training run was completed, a validation run was executed using the randomly selected 10% subset of the data that was set aside for validation. This data was not used in the training run to create the trees and train the classification model. Results for the trained model’s classification using the validation data show the model was 70% accurate with an F1-score of 50%. The validation data diagnostics reported a 93% model for the “no” HBC category and a 12% sensitivity to the “yes” HBC category. Nevertheless, the accuracy diagnostic was better. Such diagnostic considered both how well features with a particular category were predicted and how often other categories were misclassified as the category of interest. The validation data diagnostic reported a model accuracy of 78% for both the “yes” and “no” HBC categories. In terms of the validation run, the model was 70% accurate with a 40% F1-score.

## Discussion and Conclusions

The analysis of HBC from 2002 to 2022 revealed a substantial increase in western Connecticut. Most of the incidents were concentrated in western Hartford and eastern Litchfield counties. Moreover, we observed a southward trend in the location of annual mean centers and directional distributions. This was further corroborated by the emergence of several new hot spots in northeastern Fairfield and the adjacent northwestern New Haven counties. This finding offers the first published evidence supporting Evans et al.’s (2017) prediction that Connecticut’s bear population would continue to recolonize in a southern direction west of the state’s urban core.

Notably, our results, including the southern trend in monthly mean centers, suggest the distinct role of snowfall in influencing HBC. As climate change diminishes snowfall in New England, non-hibernating black bears may experience less reduced winter metabolism, potentially driving them into human settlements and triggering conflicts. The southern trend in monthly mean centers might confirm this pattern by showing that bears who live in more moderate climates, like those closer to the shoreline, have less reduced winter metabolic rates leading to HBC.

The forest-based and boosted regression also underscored the significance of forest fragmentation, distance to water, snowfall, and housing density in predicting HBC. All five significant variables exhibited nearly equal (20%) predictive power, emphasizing the prevalence of HBC in exurban landscapes. Specifically, HBC occurrence mostly overlapped with intermediate housing densities as well as near water and forested areas degraded by edge effects. Yet, contrary to our expectations, land cover did not emerge as a significant predictor. This insignificance could be attributed to the number of land cover classes (15) present in the data, which is a limitation of the software recommendation to use no more than 15 classes to avoid diminishing the model’s predictive power.

The low sensitivity for “yes” HBC is potentially related to temporal and spatial differences in HBC across the study area. Bear sightings are correlated with bear population density, and areas with higher bear populations tend to have a higher number of reported bear sightings. A greater number of bear sightings in areas within high housing density, close to fragmented forest and/or surface water have a greater likelihood of HBC. This level of granularity is not factored into our FBBC analysis due to the lack of robust bear population density data for the State of Connecticut. Therefore, we could not factor into our analysis differences in bear population density across the time period. Housing density and forest fragmentation data were not available for all years included in the analysis. Finally, it would be helpful to have spatial data on bear natural food sources.

However, we believe that the results of the FBBC analysis across the entire state of Connecticut provide valuable insights into the importance of various factors to the occurrence of HBC. Importantly, the results show good potential for conducting an entirely new study that incorporates additional data to the FBBC—not at the state level—but at larger spatial scales based on the results of the geostatistical analysis, such as the new and intensifying hot spots. Future studies could potentially use data at finer temporal and spatial scales for housing density and forest fragmentation data. Furthermore, an important consideration is for DEEP to generate robust bear population density data.

While black bears are prolific in exurban landscapes, our findings demonstrate their avoidance of urban areas and major highways, limiting their initial recolonization to western Connecticut. This is in agreement with the findings of prior studies that have stated roads as semi-permeable barriers for bears, with more trafficked major roads being less permeable (Gibeau et al. 2001; Karelus et al. 2017). Despite this, reports indicate bear presence in all 169 towns and HBC in 166 towns, including urban areas like Hartford, New Haven, and New Britain. This suggests occasional crossings during low-traffic periods or through the northern border with Massachusetts. Massachusetts’ bear population of ~45,000 is also expanding east and its established range stretches further east than Connecticut’s (Massachusetts Division of Fisheries & Wildlife, n.d.). Based on the results of our analysis, it can be surmised that eastern Connecticut is part of this recolonization process, just at a slower initial rate and with some bears entering through the northern border. Wildlife managers and residents of eastern Connecticut should anticipate an increase in HBC in the future.

Despite rigorous data quality assessments, reliance on citizen-reported HBC data introduces inherent biases and limitations. The DEEP does not verify most reports and there could be potential spatial reporting biases. Additionally, the disproportionate contribution of habituated bears to overall HBC incidents, driven by their social learning and enduring memory capabilities, poses a challenge to relying on data that generally does not identify the bear involved. Future research in this realm could explore additional socio-demographic variables such as income and incorporate more years of forest fragmentation data as it becomes available. An artificial intelligence approach could also be used to help systematically classify bear sightings as HBC. Should conflict mitigation strategies be introduced, our analysis can be modified to help investigate the efficacy of such measures.

As Connecticut’s bear population, estimated to be between 1000 and 1200, continues to expand, proactive measures from the DEEP and legislators are imperative to mitigate economic losses, protect psychological well-being, ensure human safety, and to lessen habitat fragmentation, protecting habitat for the bears. Our study highlights the potential of the RF approach in predicting HBC, paving the way for evidence-based conflict mitigation strategies. We advocate targeting the exurban areas for ethical, evidence-driven HBC mitigation strategies. Traditional approaches, such as bear hunts, appear ineffective and controversial (Gunther 1994; Zinn et al. 1998; Don Carlos et al. 2009; Treves et al. 2010; Lowery et al. 2012; Obbard et al. 2014). Recent literature has advocated for conflict mitigation and population management strategies that consider both biological and social factors, like tolerance for HBC (Organ and Ellingwood 2000; Kretser et al. 2009; Siemer et al. 2009; Bruskotter and Shelby 2010; Siemer et al. 2023). We suggest an integrated conflict mitigation approach that combines initiatives to increase public acceptance for bears as well as concrete mitigation actions, like enforcing a mandate of bear-safe trash cans and restricting bird feeders in areas prone to HBC (Lewis et al. 2015). Protecting bear habitat with conservation easements and conservation-minded land use planning might also support the natural foraging ecology of bears, in addition to supporting broader conservation goals (Beckmann and Berger 2003; Kiesecker et al. 2007; Brown et al. 2023). Wildlife corridors, such as highway underpasses, help maintain gene flow between black bear populations and can reduce roadkill incidents if properly implemented (Dixon et al. 2006; van Manen et al. 2012; Sawaya et al. 2014). Our study fills a significant knowledge gap by conducting a comprehensive long-term geospatial analysis of HBC at a statewide scale. By employing the forest-based and boosted regression approach, we offer a novel method for predicting HBC, which can inform conflict mitigation strategies in other regions facing similar challenges amidst human population growth, habitat fragmentation, and climate change. With reports of HBC on the rise in much of the United States and even other regions of the world, such as Canada, these predictive and analytical geospatial tools are crucial for promoting awareness about human–bear coexistence.

## Data Availability

The links to various datasets are listed below: Land use land cover available from https://s3-us-west-2.amazonaws.com/mrlc/NLCD_landcover_2021_release_all_files_20230630.zip. Forest fragmentation data. https://maps.cteco.uconn.edu/projects/landcover/cl-download/. Bear Sightings Data. https://portal.ct.gov/deep/online-services/online-services-home. Hydrography. https://deepmaps.ct.gov/maps/ef85cf0c55394065a8a74ea97fbd7ede/about. Town Boundaries. from https://ct-deep-gis-open-data-website-ctdeep.hub.arcgis.com/maps/82672ae5f3764021b9a4804f524f928b/about. Housing Density https://data.census.gov/table/ACSDT5Y2020.B25001?t=Housing%20Units&g=040XX00US09$1500000&y=2020. https://data.census.gov/table/DECENNIALSF12010.H2?t=Housing%20Units&g=040XX00US09$1500000&y=2010. Census Data. https://www2.census.gov/geo/pvs/tiger2010st/09_Connecticut/09/tl_2010_09_bg10.zip. https://www2.census.gov/geo/tiger/TIGER2020PL/STATE/09_CONNECTICUT/09/. Snowfall https://www.nohrsc.noaa.gov/snowfall/.
